# Depression, anxiety and medication adherence among tuberculosis patients attending treatment centres in Fako Division, Cameroon: cross-sectional study

**DOI:** 10.1192/bjo.2023.42

**Published:** 2023-04-13

**Authors:** Lionel Che Anye, Marie Ebob Agbortabot Bissong, Anna Longdoh Njundah, Joseph Nelson Siewe Fodjo

**Affiliations:** University of Buea, Buea, Cameroon; Global Health Institute, University of Antwerp, Antwerp, Belgium

**Keywords:** Tuberculosis, anxiety, depression, medication adherence, Cameroon

## Abstract

**Background:**

Tuberculosis remains a public health problem, particularly in developing countries. Patients with tuberculosis often suffer from anxiety and depression, which is likely to affect adherence to the long course of tuberculosis treatment.

**Aims:**

This study sought to investigate depression, anxiety and medication adherence among Cameroonian tuberculosis patients.

**Method:**

A cross-sectional study was conducted from March to June 2022 across five treatment centres in Fako Division, Southwest Region, Cameroon. Data were collected via face-to-face interviews with tuberculosis patients using a structured questionnaire. Sociodemographic information was obtained, and the following tools were administered to participants: the Hospital Anxiety and Depression Scale, the Oslo Social Support Scale, and the Medication Adherence Rating Scale. Multiple logistic regression models were fitted to investigate determinants of depression and anxiety.

**Results:**

A total of 375 participants were recruited (mean age: 35 ± 12.2 years; 60.5% male). The prevalence rates of depression and anxiety among tuberculosis patients were 47.7% and 29.9%, respectively. After adjusting for confounders, the odds of depression were significantly increased by having extrapulmonary tuberculosis, non-adherence to treatment, having no source of income, household size <5 and poor social support. Predictors for anxiety included extrapulmonary tuberculosis, defaulting tuberculosis treatment for ≥2 months, family history of mental illness, HIV/tuberculosis co-infection, being married, poor social support and non-adherence to treatment.

**Conclusions:**

The prevalence of depression and anxiety in tuberculosis patients is relatively high, and diverse factors may be responsible. Therefore, holistic and comprehensive care for tuberculosis patients by mental health practitioners is highly encouraged, especially for the high-risk groups identified.

## Background

Tuberculosis is a disease caused by the bacterium *Mycobacterium tuberculosis*, which is spread from person to person through the air.^1^ Generally, tuberculosis affects the human lungs, but other body parts including the brain, the kidneys or the spine can also be affected; such conditions are known as extrapulmonary tuberculosis. Despite the availability of effective antibiotherapy against tuberculosis, many patients may still die if they do not get proper and timely treatment.^2^ Notwithstanding the obtainability of affordable and effective treatment, tuberculosis still accounts for millions of active disease cases and deaths worldwide. Tuberculosis affects the poorest persons in both high-income and developing countries.^1^ According to estimates stated by the World Health Organization, tuberculosis is among the ten leading causes of death worldwide. In the past 5 years, it has occupied first place on the list of most deadly infectious diseases.^3^

The burden caused by tuberculosis is further compounded by the frequent occurrence of mental health disorders among patients, notably anxiety and depression. The fact that the routine treatment protocol for tuberculosis in Cameroon consists of a 6-month-long antibiotic course (including in-hospital isolation of tuberculosis cases during the early weeks of treatment) significantly fosters the onset of these mental health issues among patients. The presence of depressive disorders leads to poor treatment adherence, drug resistance and high rates of transmission, which put other community members at risk of being contaminated. Furthermore, depression weakens psychosocial welfare and depreciates the quality of life of affected individuals, ultimately resulting in negative treatment outcomes among tuberculosis patients.^4^ In addition, tuberculosis patients with depressive symptoms have reduced social contact and ignore social responsibilities, especially at the stage of coughing; this leads to low self-esteem and hopelessness.^5^

Previous studies report high rates of mental illness in tuberculosis patients in sub-Saharan Africa; for instance, the prevalence of depression in tuberculosis patients in Nigeria was reported to be as high as 95.5%.^6^ In Cameroon, data on this subject are relatively scarce; one study conducted in Yaoundé reported a 30.9% prevalence of depression in tuberculosis patients,^7^ whereas another study conducted in the south-west region found a prevalence of depression of 61.1% among tuberculosis patients.^8^ Another Cameroonian study among persons living with HIV (frequently associated with tuberculosis as a comorbidity) in Yaoundé found that 63% of participants had depressive symptoms.^9^ Given the paucity of information on mental illness and tuberculosis in Cameroon, we sought to investigate the prevalence and associated risk factors for depression and anxiety among tuberculosis patients, and to describe the impact on medication adherence in this population.

## Method

### Study design and setting

A facility-based multicentric cross-sectional design was employed in this study, which ran from 1 March to 30 June 2022. The study was conducted in five treatment centres in Fako Division of the English-speaking South-West region of Cameroon. Of note, this region has been subjected to civil unrest of varying intensity since 2016, owing to the anglophone crisis. The treatment centres were at Buea Regional Hospital, Mutengene Baptist Hospital, Tiko Cottage Hospital, Tiko District Hospital and the Limbe Regional Hospital. These selected hospitals are properly equipped for the diagnosis and management of tuberculosis cases. Tuberculosis treatment is generally provided free to patients in these treatment centres, being subsidised by the government. However, nursing care and hospital admission fees are paid by the patient. Of note, there were no shortages in anti-tuberculosis drugs at any of the study sites during the entire duration of the study.

### Study population and sampling

All persons of age 21 years and above who were diagnosed and confirmed with tuberculosis as per national guidelines^10,11^ and who were followed up at the selected treatment centres were included in the study. Critically ill tuberculosis patients and those with known and/or previously diagnosed mental issues were excluded from the study. A purposive sampling method was used to select the study sites (treatment centres) and to recruit participants. All tuberculosis patients who met the eligibility criteria at the selected study sites and who gave their consent to participate in the study were included. Participants were recruited following probability proportionate to size sampling whereby each study site was weighed according to its monthly patient turnover (Supplementary Appendix 1 available at https://doi.org/10.1192/bjo.2023.42).

### Sample size determination

The sample size required for this study was determined using the formula *n* = *z*^2^*p* (1 − *p*)/*e*^2^ where *p* is the prevalence, *z* is the decision variable at confidence of 95% (*z* = 1.96), and *e* is the sampling-related error risk (*e* = 0.05).^12^ We assumed a 95% confidence level falling within a 5% margin of error and a 10% non-response rate. Based on a study conducted in Walaito Sodo,^13^ in Ethiopia, a *p* = 41.5% prevalence of anxiety among tuberculosis patients, and a *p* = 43.4% prevalence of depression among tuberculosis patients were used in the calculation of the sample size. Following the sample size calculation for the two outcome variables (depression and anxiety), the larger value was taken (*n* = 397) and used as target sample size for this study.

### Data collection method and instrument

Data were collected using an open-ended structured questionnaire administered by trained nurses working at the various treatment sites. The questionnaires were divided into three sections comprising sociodemographic, clinical and psychosocial data. The data collection instrument was pretested prior to participant recruitment to ensure the reliability and validity of the tool. All interviews were conducted in English by the principal investigator (L.C.A.) and some nurses working at the study hospitals, none of whom were involved in the care or follow-up of the recruited tuberculosis patients.

The sociodemographic characteristics of the participants that were collected included age, gender, marital status, level of education, residence, income, family status, religion and employment status, whereas the clinical section included height, weight, body mass index (BMI), family history of mental illness, tuberculosis classification type (pulmonary versus extrapulmonary), duration of illness, sputum results (positive or negative sputum test for *M. tuberculosis*), treatment category (new, default, retreatment, relapse or failure), HIV comorbidity, presence of multidrug-resistant (MDR) tuberculosis and phase of treatment.

The psychosocial variables analysed were anxiety, depression, social support and medication adherence. The psychological variables anxiety and depression were assessed using the Hospital Anxiety and Depression Scale (HADS) tool as previously reported^14,15^ The HADS tool is a 14-item questionnaire commonly used to screen for anxiety and depression in patients. The 14 items in the HADS tool are separated into two seven-item subscales for anxiety (HADS-A) and depression (HADS-D). A respondent is considered to have depression or anxiety if he/she has a score of 8–10 (borderline abnormal or borderline case) or 11–21 (abnormal or case).^15^ In this study, a HADS-D or HADS-A score ≥8 was considered to indicate a case of anxiety or depression, respectively, whereas a score ≤7 was considered normal. In our study population, the HADS tool had a satisfactory Cronbach's α of 0.885.

Social support characteristics were measured using the Oslo Social Support Scale (OSSS-3), which is a brief and economic instrument to assess the level of social support received by patients.^16^ The OSSS-3 consists of three items that ask for the number of close confidants, the sense of concern from other people, and the relationship with neighbours, with a focus on the accessibility of practical help. A score from 3 to 8 was considered to indicate poor social support, and a score of 9–14 was considered to indicate strong social support.^16,17^ The social support scale had a Cronbach's α of 0.730.

Medication adherence was measured using the Medication Adherence Rating Scale (MARS).^18^ This is a ten-item self-reporting instrument which is multidimensional. It describes three dimensions of medication adherence: medication adherence behaviour (items 1–4), patients’ attitudes toward taking medication (items 5–8) and negative side-effects and attitudes towards medication (items 9–10). Questions are responded to using ’yes’ or ‘no’ on this scale. For questions 1–6 and 9–10, a ‘no’ response is indicative of good adherence, whereas for questions 7 and 8, a ‘yes’ response indicates adherence.^18^ The scores on the scale were dichotomised into non-adherence (score between 0–5) and adherence (score between 6–10).

### Data management and analysis

Data were entered into Epi Info v.26 and analysed by SPSS v.25. Continuous variables were expressed as mean and s.d., whereas categorical variables were reported as counts and percentages. Descriptive comparisons were performed using parametric and non-parametric tests as appropriate. A simple logistic regression was performed for purposefully selected explanatory variables based on the plausibility of their affecting the outcome variables (depression and anxiety). Explanatory variables with *P* ≤ 0.2 in the univariate analysis were included in the final multivariable model, in line with the cut-off *P*-value of 0.25 suggested by Bursac et al.^19^ A multiple logistic regression model was constructed, and adjusted odds ratios (AORs) with 95% confidence intervals were calculated to identify significant associations with the outcome variables (anxiety and depression). *P* < 0.05 was considered to indicate statistical significance.

### Ethical considerations

The authors assert that all procedures contributing to this work comply with the ethical standards of the relevant national and institutional committees on human experimentation and with the Helsinki Declaration of 1975, as revised in 2008. All procedures involving human subjects or patients were approved by the Institutional Review Board of the Faculty of the Health Sciences, University of Buea (no. 1657-02). Administrative authorisations were granted by the Regional Delegation of Public Health as well as the appropriate health districts and study hospitals. Participation in the study was anonymous and totally voluntary. All participants were informed about the study's aim and objectives and provided written consent to participate. No personal identifiers such as names, addresses, telephone numbers or other information identifying participants were entered into the database. Participants were informed of their right to withdraw or discontinue at any time without consequences. Confidentiality was respected at all levels of the study.

## Results

### Sociodemographic characteristics of respondents

A total of 375 respondents were enrolled into the study. Two hundred and twenty-seven (60.5%) of the respondents were males while 148 (39.5%) were females (Table 1). The mean age of the respondents was 35 years (s.d. = 12.2) years, with minimum and maximum ages of 21 years and 80 years, respectively. The majority (42.4%) of the respondents were aged 21–30 years. Regarding marital status, more than half (59.5%) of the participants were single. Only four (1.1%) participants were recorded as having no formal education. Most of the respondents (58.4%) had a monthly income of 50 000–100 000 FCFA and 27 (7.2%) had no income. A total of 224 (59.7%) respondents were urban dwellers.

**Table 1:** Sociodemographic characteristics of participants

Variable	Category	Frequency (*N*)	Percentage (%)
Treatment centre	Buea Regional Hospital	123	32.8
	Baptist Hospital Mutengene	156	41.6
	Tiko Cottage Hospital	25	6.7
	Tiko District Hospital	30	8
	Limbe Regional Hospital	41	10.9
	Overall	375	100
Sex	Female	148	39.5
	Male	227	60.5
Age, years	21–30	159	42.4
	31–40	119	31.7
	41–50	58	15.5
	51–60	16	4.3
	≥61	23	6.1
Marital status	Single	223	59.5
	Married	139	37.1
	Widow	10	2.7
	Widower	3	0.8
Educational level	Primary	104	27.7
	Secondary	212	56.5
	Tertiary	55	14.7
	No Education	4	1.1
Place of residence	Rural	151	40.3
	Urban	224	59.7
Employment status	Employed	199	53.1
	Unemployed	176	46.9
Occupation	Government employed	7	1.9
	Merchant/business	106	28.3
	Privately employed	99	26.4
	Farmer	47	12.5
	Housewife	21	5.6
	Other	95	25.3
Religion	Presbyterian	82	21.9
	Muslim	8	2.1
	Atheist	20	5.3
	Catholic	83	22.1
	Pentecostal	152	40.5
	Baptist	30	8
Living alone	No	307	81.9
	Yes	68	18.1
Family size	≤5	216	57.6
	>5	159	42.4
Monthly income (FCFA)	<50 000	37	9.9
	50 000 to 100 000	219	58.4
	100 001 to 200 000	55	14.7
	200 001 to 300 000	23	6.1
	>300 000	14	3.7
	No income	27	7.2

### Clinical and psychosocial characteristics of the respondents

Of the respondents 325 (86.7%) had been diagnosed with pulmonary tuberculosis (Table 2). Most of the respondents (342, 91.2%) were in the new tuberculosis treatment category. Two hundred and eighteen (58.1%) were in the continuation phase of tuberculosis treatment. More than half of the respondents (88.8%) were out-patients, coming for follow-up treatment appointments. The majority, 263 (70.1%), reported having strong social support from their family and community. Good medication adherence was noted in 340 (90.7%) of participants.

**Table 2:** Clinical and social support findings

Variable	Category	Frequency (*N*)	Percentage (%)
Tuberculosis classification type	Pulmonary tuberculosis	325	86.7
	Extrapulmonary tuberculosis	50	13.3
Sputum type	Positive	296	78.9
	Negative	79	21.1
Category of treatment	New	342	91.2
	Default	11	2.9
	Retreatment	16	4.3
	Relapse	3	0.8
	Failure	3	0.8
HIV/tuberculosis co-infection	No	299	79.7
	Yes	76	20.3
Multidrug-resistant tuberculosis	No	370	98.7
	Yes	5	1.3
Phase of treatment	Initiation phase	157	41.9
	Continuation phase	218	58.1
Patient status	Admitted to hospital	42	11.2
	Out-patient	333	88.8
Substance use	No	318	84.8
	Yes	57	15.2
Family history of mental illness	No	359	95.7
	Yes	16	4.3
Body mass index	Normal	164	43.7
	Underweight	49	13.1
	Overweight	43	11.5
	Obese	21	5.6
	Unknown	98	26.1
Duration of illness	≤4 weeks	110	29.3
	>4 weeks	265	70.7
Medication adherence	Yes	340	90.7
	No	35	9.3
Level of social support	Poor	112	29.9
	Strong	263	70.1

### Prevalence of depression and anxiety among tuberculosis patients

The prevalence of depression in our study population (HADS-D score >7) was 47.7% (95% CI: 42.6–52.9), whereas that of anxiety (HADS-A score >7) was 29.9% (95% CI: 25.3–34.8). The gender-specific prevalence rates of depression and anxiety were higher among male (59.2% and 54.5%, respectively) compared with female participants (40.8% and 45.5%, respectively).

### Factors associated with depression and anxiety

Cross-tabulation using a chi-squared test showed a significant relationship between depression and the following factors: hospital (site of study) (*P* < 0.001), educational level (*P* < 0.011), medication adherence (*P* = 0.001), level of social support (*P* = 0.031), classification type of tuberculosis (*P* < 0.001), patient status (*P* = 0.001), BMI (*P* < 0.001), HIV/tuberculosis co-infection (*P* = 0.047) and MDR tuberculosis (*P* < 0.001); whereas the following factors had a significant relationship with anxiety: treatment centres (*P* < 0.001), employment status (*P* = 0.009), monthly income (*P* < 0.001), classification type of tuberculosis (*P* = 0.019), medication adherence (*P* = 0.001), level of social support (*P* = 0.001), patient status (*P* = 0.001) and BMI (*P* < 0.027); see Supplementary Appendix 2.

### Factors associated with depression among tuberculosis patients

After adjusting for confounders, several variables were found to be independently associated with depression (Table 3). Compared with tuberculosis patients attending the Buea Regional Hospital, those from the Baptist Hospital Mutengene, Tiko District Hospital and Limbe Regional Hospital were less likely to be depressed (AOR < 1). Having a household size greater than five and receiving stronger social support were found to be protective factors against depression (AOR < 1). Conversely, those with extrapulmonary tuberculosis and those with no income had significantly higher odds of being depressed (AOR > 1).

**Table 3:** Multivariable logistic regression for depression and associated risk factors among tuberculosis patients

Variables	Category	Depression		Crude OR (95% CI)	Adjusted OR (95% CI)	*P*-value
		Yes, *N* (%)	No, *N* (%)			
Treatment centre	Buea Regional Hospital	83 (67.5)	40 (32.5)	1	1	
	Baptist Hospital Mutengene	62 (39.8)	94 (60.2)	0.65 (0.47, 0.9)	0.37 (0.18, 0.77)	0.009**
	Tiko Cottage Hospital	10 (40)	15 (60)	0.66 (0.29, 1.48)	0.36 (0.08, 1.53)	0.169
	Tiko District Hospital	6 (20)	24 (80)	0.25 (0.1, 0.61)	0.04 (0, 0.18)	0.000**
	Limbe Regional Hospital	18 (44)	23 (56)	0.78 (0.42, 1.45)	0.21 (0.06, 0.67)	0.009**
Sex	Female	73 (49.3)	75 (50.7)	1	1	
	Male	106 (46.7)	121 (53.3)	0.88 (0.68, 1.14)	0.71 (0.37, 1.36)	0.309
Education	Primary	63 (60.6)	41 (39.4)	1	1	
	Secondary	90 (42.5)	122 (57.5)	0.73 (0.56, 0.96)	0.39 (0.18, 0.86)	0.020*
	Tertiary	23 (41.8)	32 (58.2)	0.71 (0.42, 1.22)	0.42 (0.14, 1.25)	0.121
	No education	3 (75.0)	1 (25.0)	2.99 (0.31, 28.84)	1.98 (0.1, 38.45)	0.650
Residence	Rural	74 (49.0)	77 (51.0)	1	1	
	Urban	105 (46.9)	119 (53.1)	0.88 (0.67, 1.14)	0.74 (0.38, 1.44)	0.385
Employment	Employed	103 (51.8)	96 (48.2)	1	1	
	Unemployed	76 (43.2)	100 (56.8)	0.76 (0.56, 1.02)	0.72 (0.34, 1.54)	0.405
Family size	≤5	111 (51.4)	105 (48.6)	1	1	
	>5	68 (42.8)	91 (57.2)	0.74 (0.54, 1.02)	0.52 (0.28, 0.95)	0.035*
Income	<50 000	10 (27.0)	27 (73.0)	1	1	
	50 000 to 100 000	111 (50.7)	108 (49.3)	1.02 (0.78, 1.33)	2.76 (0.84, 9.05)	0.093
	100 001 to 200 000	27 (49.1)	28 (50.9)	0.96 (0.56, 1.63)	2.5 (0.64, 9.7)	0.183
	200 001 to 300 000	11 (47.8)	12 (52.2)	0.91 (0.4, 2.07)	5.22 (1.07, 25.3)	0.040*
	>300 000	6 (42.9)	8 (57.1)	0.75 (0.26, 2.16)	2.14 (0.3, 15.14)	0.445
	No income	14 (51.9)	13 (48.1)	1.07 (0.5, 2.29)	4.88 (1.18, 20.11)	0.028*
Classification type	Pulmonary tuberculosis	141 (43.4)	184 (56.6)	1	1	
	Extrapulmonary tuberculosis	38 (76)	12 (24.0)	3.16 (1.65, 6.05)	5.89 (1.89, 18.34)	0.002**
Sputum type	Positive	131 (44.3)	165 (55.7)	1	1	
	Negative	48 (60.8)	31 (39.2)	0.8 (0.65, 1)	0.54 (0.21, 1.38)	0.201
Patient status	Admitted to hospital	30 (71.5)	12 (28.5)	1	1	
	Out-patient	149 (44.8)	184 (55.2)	0.58 (0.36, 0.91)	0.27 (0.09, 0.81)	0.020*
Substance use (cigarette and/or alcohol use)	No	151 (47.5)	167 (52.5)	1	1	
	Yes	28 (49.2)	29 (50.8)	0.96 (0.57, 1.62)	1.25 (0.49, 3.17)	0.635
Family history of mental illness	No	170 (47.4)	189 (52.6)	1	1	
	Yes	9 (56.3)	7 (43.7)	1.28 (0.47, 3.45)	1.49 (0.39, 5.73)	0.555
HIV/tuberculosis co-infection	No	135 (45.2)	164 (54.8)	1	1	
	Yes	44 (57.9)	32 (42.1)	1.37 (0.87, 2.16)	1.77 (0.8, 3.92)	0.158
Phase of treatment	Initiation phase	79 (50.4)	78 (49.6)	1	1	
	Continuation phase	100 (45.9)	118 (54.1)	0.84 (0.64, 1.1)	1.08 (0.47, 2.47)	0.847
Body mass index	Normal	72 (44)	92 (56)	1	1	
	Underweight	32 (65.4)	17 (34.6)	1.88 (1.04, 3.38)	2.43 (0.92, 6.42)	0.073
	Overweight	10 (23.3)	33 (76.7)	0.3 (0.14, 0.61)	0.23 (0.08, 0.65)	0.006**
	Obese	14 (66.7)	7 (33.3)	2 (0.8, 4.95)	1.85 (0.47, 7.29)	0.375
	Unknown	51 (52.1)	47 (47.9)	1.08 (0.73, 1.61)	1.11 (0.55, 2.26)	0.761
Illness duration	≤4 weeks	56 (50.9)	54 (49.1)	1	1	
	>4 weeks	123 (46.4)	142 (53.6)	0.86 (0.68, 1.1)	1.31 (0.54, 3.16)	0.539
Level of social support	Poor social support	63 (56.3)	49 (43.7)	1	1	
	Strong social support	116 (44.2)	147 (55.8)	0.78 (0.61, 1)	0.3 (0.15, 0.61)	0.001**
Medication adherence	Adherent	153 (45)	187 (55)	1	1	
	Non-adherent	26 (74.3)	9 (25.7)	2.88 (1.35, 6.16)	3.14 (1.28, 7.67)	0.012*

*P < 0.05; **P < 0.01.

### Factors associated with anxiety among tuberculosis patients

After adjusting for confounders, the odds of having anxiety were 7.506 times higher among extrapulmonary tuberculosis patients (*P* = 0.004, AOR = 7.506, 95% CI: 1.880–29.968) than in pulmonary tuberculosis patients (Table 4). Compared with tuberculosis patients attending the Buea Regional Hospital, patients from all other hospitals had significantly lower odds of having anxiety. Being unemployed and receiving strong social support were also associated with reduced odds for anxiety. By contrast, non-adherence to treatment, HIV/tuberculosis co-infection and family history of mental illness significantly increased the odds of anxiety among the participants.

**Table 4:** Multivariable logistic regression for anxiety and associated risk factors among tuberculosis patients

Variables	Category	Anxiety		Crude OR (95% CI)	Adjusted OR (95% CI)	*P*-value
		Yes, *N* (%)	No, *N* (%)			
Hospital	Buea Regional Hospital	67 (54.5)	56 (45.5)	1	1	
	Baptist Hospital Mutengene	28 (18)	128 (82)	0.22 (0.145, 0.329)	0.16 (0.07, 0.4)	0.000**
	Tiko Cottage Hospital	4 (16)	21 (84)	0.19 (0.065, 0.555)	0.02 (0, 0.26)	0.002**
	Tiko District Hospital	8 (26.7)	22 (73.3)	0.36 (0.162, 0.817)	0.06 (0.01, 0.32)	0.001**
	Limbe Regional Hospital	5 (12.2)	36 (87.8)	0.14 (0.055, 0.354)	0.01 (0, 0.06)	0.000**
Age, years	21–30	46 (29)	113 (71)	1	1	
	31–40	29 (24.4)	90 (75.6)	0.32 (0.212, 0.49)	0.25 (0.08, 0.74)	0.013*
	41–50	24 (41.4)	34 (58.6)	0.71 (0.419, 1.19)	0.66 (0.18, 2.38)	0.527
	51–60	6 (37.5)	10 (62.5)	0.6 (0.218, 1.651)	0.33 (0.05, 2.16)	0.249
	≥61	7 (30.5)	16 (69.5)	0.44 (0.18, 1.063)	0.43 (0.07, 2.48)	0.346
Sex	Female	51 (34.5)	97 (65.5)	1	1	
	Male	61 (26.9)	166 (73.1)	0.37 (0.274, 0.493)	0.39 (0.18, 0.87)	0.022*
Marital status	Single	56 (25.2)	167 (74.8)	1	1	
	Married	50 (36)	89 (64)	0.56 (0.397, 0.794)	3.4 (1.19, 9.71)	0.022*
	Widow	4 (40)	6 (60)	0.67 (0.188, 2.362)	0.61 (0.06, 5.85)	0.673
	Widower	2 (66.7)	1 (33.3)	2 (0.181, 22.056)	2.32 (0.06, 81.14)	0.642
Education	Primary	37 (35.6)	67 (64.4)	1	1	
	Secondary	55 (26)	157 (74)	0.35 (0.258, 0.476)	0.58 (0.22, 1.52)	0.276
	Tertiary	19 (34.6)	36 (65.4)	0.53 (0.303, 0.92)	0.6 (0.17, 2.08)	0.424
	No education	1 (25)	3 (75)	0.33 (0.035, 3.205)	6.05 (0.24, 147.17)	0.269
Residence	Rural	45 (29.9)	106 (70.1)	1	1	
	Urban	67 (30)	157 (70)	0.43 (0.321, 0.568)	1.46 (0.66, 3.21)	0.341
Employment	Employed	71 (35.7)	128 (64.3)	1	1	
	Unemployed	41 (23.3)	135 (76.7)	0.30 (0.214, 0.431)	0.17 (0.06, 0.46)	0.000**
Occupation	Government employed	6 (85.8)	1 (14.2)	1	1	
	Merchant/business	31 (29.3)	75 (70.7)	0.41 (0.272, 0.628)	0.11 (0, 1.93)	0.132
	Privately employed	35 (35.4)	64 (64.6)	0.55 (0.362, 0.826)	0.16 (0, 2.78)	0.209
	Farmer	15 (32)	32 (68)	0.47 (0.254, 0.866)	0.19 (0.01, 3.45)	0.265
	Housewife	5 (23.9)	16 (76.1)	0.31 (0.114, 0.853)	0.07 (0, 1.75)	0.108
	Other	20 (21.1)	75 (78.9)	0.27 (0.163, 0.437)	0.13 (0, 2.79)	0.193
Living alone	No	98 (32)	209 (68)	1	1	
	Yes	14 (20.6)	54 (79.4)	0.26 (0.144, 0.467)	0.46 (0.16, 1.28)	0.140
Family size	≤5	63 (29.2)	153 (70.8)	1	1	
	>5	49 (30.9)	110 (69.1)	0.45 (0.318, 0.624)	0.85 (0.41, 1.78)	0.683
Income	<50 000	11 (29.8)	26 (70.2)	1	1	
	50 000 to 100 000	60 (27.4)	159 (72.6)	0.38 (0.28, 0.508)	0.19 (0.05, 0.7)	0.013*
	100 001 to 200 000	21 (38.2)	34 (61.8)	0.62 (0.359, 1.064)	0.59 (0.13, 2.71)	0.505
	200 001 to 300 000	2 (8.7)	21 (91.3)	0.09 (0.022, 0.406)	0.03 (0, 0.37)	0.006**
	>300 000	11 (78.6)	3 (21.4)	3.67 (1.023, 13.143)	6.23 (0.61, 63.69)	0.122
	No Income	7 (26)	20 (74)	0.35 (0.148, 0.828)	0.79 (0.15, 3.96)	0.776
Classification type	Pulmonary tuberculosis	90 (27.7)	235 (72.3)	1	1	
	Extrapulmonary tuberculosis	22 (44)	28 (56)	0.77 (0.45, 1.373)	7.5 (1.87, 29.96)	0.004**
Sputum type	Positive	83 (28.1)	213 (71.9)	1	1	
	Negative	29 (36.8)	50 (63.2)	0.58 (0.367, 0.916)	0.35 (0.1, 1.21)	0.099
Patient status	Admitted to hospital	22 (52.4)	20 (47.6)	1	1	
	Out-patient	90 (27.1)	243 (72.9)	0.37 (0.291, 0.472)	0.53 (0.16, 1.77)	0.307
Treatment category	New	96 (28.1)	246 (71.9)	1	1	
	Default	6 (54.6)	5 (45.4)	1.2 (0.366, 3.932)	12.68 (1.67, 95.98)	0.014*
	Retreatment	8 (50)	8 (50)	1 (0.375, 2.664)	2.23 (0.49, 10)	0.292
	Relapse	1 (33.4)	2 (66.6)	0.5 (0.045, 5.514)	17.44 (0.74, 408.14)	0.075
	Failure	1 (33.4)	2 (66.6)	0.5 (0.045, 5.514)	0.19 (0, 117.52)	0.619
Substance use (cigarette and/or alcohol use).	No	93 (29.3)	225 (70.7)	1	1	
	Yes	19 (33.4)	38 (66.6)	0.5 (0.288, 0.867)	2.06 (0.55, 7.67)	0.277
Family history of mental illness	No	104 (29)	255 (71)	1	1	
	Yes	8 (50)	8 (50)	1 (0.375, 2.664)	8.85 (1.96, 39.89)	0.005**
HIV/tuberculosis co-infection	No	85 (28.5)	214 (71.5)	1	1	
	Yes	27 (35.6)	49 (64.4)	0.55 (0.344, 0.881)	4.14 (1.61, 10.63)	0.003**
Multidrug-resistant tuberculosis	No	110 (29.8)	260 (70.2)	1	1	
	Yes	2 (40)	3 (60)	0.67 (0.111, 3.99)	2.84 (0.12, 62.62)	0.508
Level of social support	Poor	47 (42)	65 (58)	1	1	
	Strong	65 (24.8)	198 (75.2)	0.33 (0.248, 0.434)	0.28 (0.12, 0.64)	0.003**
Medication adherence	Adherent	93 (27.4)	247 (72.6)	1	1	
	Non-adherent	19 (54.3)	16 (45.7)	1.19 (0.611, 2.309)	3.46 (1.58, 7.59)	0.002**

*P < 0.05; **P < 0.01.

## Discussion

There have been few studies that have assessed the burden of depression and anxiety among tuberculosis patients globally and in Cameroon in particular. This study fills this gap by providing such data in the Cameroonian context and exploring how this affects adherence to tuberculosis treatment in selected hospitals. Overall, mental health findings among participants differed significantly by hospital, with tuberculosis patients followed up at the Buea Regional Hospital having the highest odds for both anxiety and depression. Whether these differences are related to civil unrest or other town-specific elements remains to be confirmed. Further research is warranted to understand these disparities and improve tuberculosis care in these health facilities.

### Prevalence of depression and associated factors among tuberculosis patients

On comparison with the evidence available in the literature, the prevalence of depression among tuberculosis patients in our study (47.4%) was similar to the 46.3% observed in Pakistan^20^ and the 43.4% observed in Wailoto South Ethiopia, also obtained using the HADS tool.^13^ Other studies that used HADS for depression screening found higher values, notably in Southwest Ethiopia (55.9%)^5^ and Turkey (60.5%).^21^ The observed discrepancy might be due in part to differences in the study settings and populations. Other studies that used different tools to identify cases of depression also found varied prevalence rates in south-west Cameroon (61.1%),^8^ Nigeria (48.6%)^22^ and Ethiopia (19.8%,^23^ 31.1%,^24^ 54.0%,^25^ 51.9%^26^), and Pakistan (56%).^27^ These results are hardly comparable with ours owing to the different methodologies used to assess outcomes.

The observation in our study that strong social support has antidepressive effects, similar to findings from other studies,^19,25,26^ attests to the paramount need for families and relatives to be involved in the care of tuberculosis patients. Indeed, participants in our study that were treated as out-patients were less likely to become depressed because their family environment constituted a firm support system. Poor social support gives patients feelings of neglect, isolation and worthlessness, and strong social support is vital for the prevention of such feelings.^28^ Therefore, sensitisation campaigns could be organised in target communities to emphasise the benefits of social support for the well-being of patients. At the health facility level, tuberculosis care programmes should integrate family sessions to encourage relatives to support the tuberculosis patient throughout the treatment process.

Unlike previous studies carried out in Ethiopia and the Philippines^24,29,30^ in which respondents with low BMI (underweight) had higher odds of being depressed, in this study we identified overweight as being protective against depression (AOR = 0.231, 95% CI: 0.081–0.658). From a cultural perspective in Cameroon, being overweight gives a sense of well-being and good living, which makes people more cheerful about themselves and therefore less depressed. This is in contrast with patients diagnosed with extrapulmonary tuberculosis, who have greater odds of depression because of the bleaker prognosis of this condition compared with pulmonary tuberculosis. Therefore, more emphatic and targeted psychosocial interventions may need to be specially designed for this particular group of tuberculosis patients, and closer and/or more frequent monitoring of their well-being may be necessary during follow-up.

An intriguing finding was that participants’ income levels were significantly associated with depression: persons with no income and those with incomes above 50 000 FCFA per month had higher odds for depression compared with persons who earned 50 000 FCFA monthly. Although the full explanation for this observation may be complex, it is plausible that tuberculosis made those with no income even more depressed as they had to rely on others not just for provision but also for medical care. Conversely, those earning above 50 000 FCFA may suffer substantial losses of income during the several months of tuberculosis treatment as they would be unable to work during that period. Thus, professional counselling and rehabilitation are important aspects to consider during the tuberculosis treatment process, as such interventions may improve patients’ mood and give them hope of possible financial restoration after recovery.

### Prevalence of anxiety and associated factors among tuberculosis patients

Overall, 29.9% of our participants had anxiety. These findings were similar to those from obtained in China (prevalence of 29.3%^29^) and in Brazil (prevalence of 26.0%^21^) but lower than those from Ethiopia (41.5%,^13^ 54.6%^5^) and Pakistan (47.2%),^20^ all of which were obtained using the HADS tool. The variation might be due to the differences in the study setting and population variations. In our study, being male was a protective factor with respect to anxiety. Different studies have also shown that anxiety disorders are more common in females than males. Differences in biological factors such as menstruation and social factors such as the burden of household responsibilities may contribute to the higher prevalence of anxiety among females than males.^5,21,28,31^ This finding certainly warrants a gender-specific approach to the management of tuberculosis patients, as different sexes are affected differently by the disease. In addition, marital status should be considered when treating tuberculosis patients, as we found that married patients were more likely to develop anxiety. This could be due to an inability to work as usual and care for the family, and/or the patient's fear of contaminating his/her spouse and child(ren) with the disease. These patients and their families would very much benefit from proper counselling and guidance in this regard.

Our study is not the first to report that HIV and tuberculosis co-infection is anxiogenic.^32^ Indeed, HIV/tuberculosis co-infected patients experience a compounded risk for common mental disorders because of the social stigma and discrimination associated with each of those conditions.^33^ Moreover, the fact that they have a poorer prognosis fuels their uncertainties concerning the future. Therefore, they require close attention and follow-up of their mental health. The increased psychosocial needs of this co-infected population should be taken into account at treatment units for both HIV and tuberculosis.

As observed with depression, strong social support was associated with lower odds for anxiety. It has been demonstrated that good social support is a key component in the prevention of anxiety among healthy individuals,^34^ and this is particularly so among tuberculosis patients. In view of the fact that social support significantly affects both anxiety and depression in tuberculosis patients, we recommend that tuberculosis treatment centres should invest in facilitating daily communication between tuberculosis patients undergoing hospital treatment and their families and friends. Moreover, counselling families and communities about the importance of providing support to tuberculosis patients when they return home would go a long way towards improving the well-being of the latter.

### Adherence to tuberculosis treatment by patients in Fako division of Cameroon

According to the MARS, 90.7% of tuberculosis patients among our respondents were adherent to treatment, whereas 9.3% were non-adherent. The high adherence in our setting could be due to increased awareness created by the medical staff on the importance of adhering to the treatment protocol, the free provision of medications at no cost to the patient and the fact that patients were aware that taking the medication would improves their treatment outcomes. Such awareness among tuberculosis patients seems to be a determinant of medication adherence and should be preferred to other methods such as daily SMS reminders, which have not proved effective in increasing tuberculosis treatment adherence in Cameroon.^35^ A study in South Africa found a slightly lower adherence rate (76%) among tuberculosis patients compared with that of our study.^36^ Furthermore, our findings also showed a higher adherence to tuberculosis treatment compared with data from Ethiopia, where studies suggested a pooled prevalence of non-adherence of about 20% (corresponding to an 80% adherence rate), possibly owing to stigma, high cost of drugs, lack of social support, disrespect from healthcare professionals, health system factors and non-accessibility of drugs.^37^

### Associations of depression and anxiety with treatment adherence

Both depression and anxiety were found to be positively associated with non-adherence to tuberculosis treatment. This is because non-adherence leads to poor prognosis and thus poor treatment outcomes, leading to breakdown of the mental health of tuberculosis patients. This is in line with a meta-analysis study in which depressive symptoms in tuberculosis patients were associated with poor medication adherence.^38^ Given that anxiety and depression are strong determinants of treatment adherence in tuberculosis, they could be targeted by interventions aiming at attaining 100% tuberculosis treatment adherence in Cameroon. We therefore recommend that mental healthcare should become an integral part of tuberculosis patient management to ensure optimal outcomes. This could be achieved by training the existing health professionals involved in tuberculosis care (mostly nurses) in mental health screening and initial management of abnormalities before referral to a specialist if need be. Such a model that capitalises on the skills of non-physicians was previously suggested for epilepsy care in similar settings,^39^ with important policy implications which could be translated into tuberculosis management schemes. At the family and community level, modifiable factors associated with anxiety and depression (e.g. social support) could be leveraged to foster better adherence during tuberculosis treatment.

### Strengths and limitations of the study

In this study, standard tools were used to assess the associated variables, perceived level of social support, and MARS scores. HADS was used to measure the outcome variables, depression and anxiety. In addition, this study addressed potential confounding variables including medication adherence and participants’ income, which were not considered in most previous studies. Furthermore, the study was multicentric, drawing participants from multiple sites. As such the results, can be generalised to tuberculosis patients being followed up in similar settings, notably in Cameroon and even sub-Saharan Africa, particularly those in their initial weeks of treatment.

However, the study had some limitations. Only the probable prevalence of anxiety and depression was assessed, as we used a screening tool rather than a diagnostic tool. There was a possibility of social desirability bias, as the study was conducted using face-to-face interviews and involved some nurses working in the same hospital where the tuberculosis patients were being followed up. We also had plausibility of recall bias as some of the factors were assessed based on history. Furthermore, important factors including perceived severity of tuberculosis symptoms, perceived stigma and stress levels of tuberculosis patients were not addressed in this study. The expected sample size was 397, but we effectively interviewed only 375 (94.45% of the target sample), which may have underpowered the study for some comparisons.

### Implications

Of the participants in this study, 47.7% and 29.9% were found to have depression and anxiety, respectively. Income levels, level of social support, patient status and classification of tuberculosis were the factors that were significantly associated with both depression and anxiety. This highlights the need to pay attention to the mental health conditions of patients with tuberculosis, particularly those with identified risk factors. Tuberculosis treatment centres should develop guidelines to screen and treat depression and anxiety among tuberculosis patients, and possibly other presenting mental disorders. Additional research on determinants of depression and anxiety among tuberculosis patients should be conducted to strengthen and build on the findings of the current study.

## Data Availability

The data presented in this paper are freely available from the authors upon reasonable request.

## Supplementary material

For supplementary material accompanying this paper visit http://doi.org/10.1192/bjo.2023.42. click here to view supplementary material
