# Supplementary material for: Depression, anxiety and medication adherence among tuberculosis patients attending treatment centres in Fako Division, Cameroon: cross-sectional study
Source: BJPsych Open. 2023 Apr 13;9(3):e65. doi: 10.1192/bjo.2023.42 (PMC10134253; doi:10.1192/bjo.2023.42)
Supplement: Supplementary file 1 [file bjosup.zip › S205647242300042Xsup001.docx]

**Supplementary Appendix 1**

**Article: *Depression, Anxiety, and Medication Adherence amongst Tuberculosis Patients attending treatment centres in Fako Division, Cameroon: A Cross-sectional study***

**Distribution of Sample size according to Probability Proportionate to Size Sampling**

| **Probability Proportionate to Size Sampling across the Study sites** | | | | | | |
| --- | --- | --- | --- | --- | --- | --- |
| **Hospital** | **Average Monthly TB Patient Turn out** | **% Above 21 years of age** | **Number of Cases aged ≥ 21yrs** | **Proportions** | **Expected Sample Size** | **Recruited sample size** |
| Buea Regional Hospital | 35 | 90% | 32 | 0.31 | 123 | 123 |
| Baptist Hospital Mutengene | 48 | 94% | 45 | 0.44 | 174 | 156 |
| Tiko Cottage Hospital | 8 | 90% | 7 | 0.07 | 27 | 25 |
| Tiko District Hospital | 8 | 90% | 7 | 0.07 | 27 | 30 |
| Limbe Regional Hospital | 14 | 90% | 12 | 0.12 | 46 | 41 |
| **Total** | **112** |  | **102** | **1** | **397** | **375** |
