# Supplementary material for: Depression, anxiety and medication adherence among tuberculosis patients attending treatment centres in Fako Division, Cameroon: cross-sectional study
Source: BJPsych Open. 2023 Apr 13;9(3):e65. doi: 10.1192/bjo.2023.42 (PMC10134253; doi:10.1192/bjo.2023.42)
Supplement: Supplementary file 1 [file bjosup.zip › S205647242300042Xsup002.docx]

**Supplementary Appendix 2**

**Article: *Depression, Anxiety, and Medication Adherence amongst Tuberculosis Patients attending treatment centres in Fako Division, Cameroon: A Cross-sectional study***

**Table S2.1: Association of depression with sociodemographic factors amongst TB patients in the Fako Division of Cameroon**

| **Variable** | **Variable Response** | **Severity of Depression** | | | **P-Value** |
| --- | --- | --- | --- | --- | --- |
|  |  | **Normal** | **Borderline Abnormal** | **Abnormal** |  |
|  |  | **N (%)** | **N (%)** | **N (%)** |  |
| Name of Hospital | Buea Regional Hospital | 40 (32.5) | 44 (35.8) | 39 (31.7) | <0.001 |
|  | Baptist Hospital Mutengene | 94 (60.3) | 50 (32.1) | 12 (7.7) |  |
|  | Tiko Cottage Hospital | 15 (60) | 3 (12) | 7 (28) |  |
|  | Tiko District Hospital | 24 (80) | 2 (6.7) | 4 (13.3) |  |
|  | Limbe Regional Hospital | 23 (56.1) | 13 (31.7) | 5 (12.2) |  |
| Age group (years) | 21-30 | 92 (57.9) | 38 (23.9) | 29 (18.2) | 0.091 |
|  | 31-40 | 63 (52.9) | 40 (33.6) | 16 (13.4) |  |
|  | 41-50 | 24 (41.4) | 18 (31) | 16 (27.6) |  |
|  | 51-60 | 9 (56.3) | 6 (37.5) | 1 (6.3) |  |
|  | >=61 | 8 (34.8) | 10 (43.5) | 5 (21.7) |  |
| Sex of respondents | Female | 75 (50.7) | 51 (34.5) | 22 (14.9) | 0.215 |
|  | Male | 121 (53.3) | 61 (26.9) | 45 (19.8) |  |
| Marital Status | Single | 120 (53.8) | 65 (29.1) | 38 (17) | 0.157 |
|  | Married | 72 (51.8) | 44 (31.7) | 23 (16.5) |  |
|  | Widow | 3 (30) | 3 (30) | 4 (40) |  |
|  | Widower | 1 (33.3) | 0 (0) | 2 (66.7) |  |
| Educational Level | Primary | 41 (39.4) | 38 (36.5) | 25 (24) | 0.001 |
|  | Secondary | 122 (57.5) | 63 (29.7) | 27 (12.7) |  |
|  | Tertiary | 32 (58.2) | 11 (20) | 12 (21.8) |  |
|  | No Education | 1 (25) | 0 (0) | 3 (75) |  |
| Place of Residence | Rural | 77 (51) | 43 (28.5) | 31 (20.5) | 0.537 |
|  | Urban | 119 (53.1) | 69 (30.8) | 36 (16.1) |  |
| Employment Status | Employed | 96 (48.2) | 64 (32.2) | 39 (19.6) | 0.25 |
|  | Unemployed | 100 (56.8) | 48 (27.3) | 28 (15.9) |  |
| Occupation | Government Employed | 0 (0) | 1 (14.3) | 6 (85.7) | <0.001 |
|  | Merchant/Business | 50 (47.2) | 38 (35.8) | 18 (17) |  |
|  | Private Employed | 53 (53.5) | 29 (29.3) | 17 (17.2) |  |
|  | Farmer | 25 (53.2) | 8 (17) | 14 (29.8) |  |
|  | Housewife | 11 (52.4) | 7 (33.3) | 3 (14.3) |  |
|  | Others | 57 (60) | 29 (30.5) | 9 (9.5) |  |
| Religion | Presbyterian | 33 (40.2) | 20 (24.4) | 29 (35.4) | <0.001 |
|  | Muslim | 5 (62.5) | 3 (37.5) | 0 (0) |  |
|  | Atheist | 11 (55) | 7 (35) | 2 (10) |  |
|  | Catholic | 51 (61.4) | 22 (26.5) | 10 (12) |  |
|  | Pentecostals | 76 (50) | 57 (37.5) | 19 (12.5) |  |
|  | Baptist | 20 (66.7) | 3 (10) | 7 (23.3) |  |
| Living alone | No | 163 (53.1) | 95 (30.9) | 49 (16) | 0.115 |
|  | Yes | 33 (48.5) | 17 (25) | 18 (26.5) |  |
| Family Size Groups | <=5 | 105 (48.6) | 75 (34.7) | 36 (16.7) | 0.057 |
|  | >5 | 91 (57.2) | 37 (23.3) | 31 (19.5) |  |
| Monthly Income | <50 000 | 27 (73) | 3 (8.1) | 7 (18.9) | 0.012 |
|  | 50 000 to 100 000 | 108 (49.3) | 63 (28.8) | 48 (21.9) |  |
|  | 100 001 to 200 000 | 28 (50.9) | 19 (34.5) | 8 (14.5) |  |
|  | 200 001 to 300 000 | 12 (52.2) | 11 (47.8) | 0 (0) |  |
|  | >300 000 | 8 (57.1) | 4 (28.6) | 2 (14.3) |  |
|  | No Income | 13 (48.1) | 12 (44.4) | 2 (7.4) |  |

**Table S2.2: Association of depression with Clinical, social support and medication adherence factors amongst TB patients in the Fako Division of Cameroon.**

| **Variable** | **Variable Response** | **Severity of Depression** | | | **P-Value** |
| --- | --- | --- | --- | --- | --- |
|  |  | **Normal** | **Borderline Abnormal** | **Abnormal** |  |
|  |  | **N (%)** | **N (%)** | **N (%)** |  |
| Classification Type | Pulmonary TB | 184 (56.6) | 86 (26.5) | 55 (16.9) | <0.001 |
|  | Extrapulmonary TB | 12 (24) | 26 (52) | 12 (24) |  |
| Sputum Type | Positive | 165 (55.7) | 83 (28) | 48 (16.2) | 0.031 |
|  | Negative | 31 (39.2) | 29 (36.7) | 19 (24.1) |  |
| Patient Status | Hospitalized | 12 (28.6) | 16 (38.1) | 14 (33.3) | 0.002 |
|  | Outpatient | 184 (55.3) | 96 (28.8) | 53 (15.9) |  |
| Category of Treatment | New | 178 (52) | 103 (30.1) | 61 (17.8) | 0.832 |
|  | Default | 5 (45.5) | 4 (36.4) | 2 (18.2) |  |
|  | Retreatment | 8 (50) | 4 (25) | 4 (25) |  |
|  | Relapse | 3 (100) | 0 (0) | 0 (0) |  |
|  | Failure | 2 (66.7) | 1 (33.3) | 0 (0) |  |
| Substance Use | No | 167 (52.5) | 101 (31.8) | 50 (15.7) | 0.019 |
|  | Yes | 29 (50.9) | 11 (19.3) | 17 (29.8) |  |
| Family History of Mental Illness | No | 189 (52.6) | 110 (30.6) | 60 (16.7) | 0.017 |
|  | Yes | 7 (43.8) | 2 (12.5) | 7 (43.8) |  |
| TB/HIV Coinfection | No | 164 (54.8) | 81 (27.1) | 54 (18.1) | 0.057 |
|  | Yes | 32 (42.1) | 31 (40.8) | 13 (17.1) |  |
| MDR TB CASE | No | 196 (53) | 110 (29.7) | 64 (17.3) | 0.02 |
|  | Yes | 0 (0) | 2 (40) | 3 (60) |  |
| Phase of treatment | Initiation Phase | 78 (49.7) | 40 (25.5) | 39 (24.8) | 0.009 |
|  | Continuation Phase | 118 (54.1) | 72 (33) | 28 (12.8) |  |
| BMI | Normal | 92 (56.1) | 51 (31.1) | 21 (12.8) | <0.001 |
|  | Underweight | 17 (34.7) | 12 (24.5) | 20 (40.8) |  |
|  | Overweight | 33 (76.7) | 7 (16.3) | 3 (7) |  |
|  | Obesity | 7 (33.3) | 10 (47.6) | 4 (19) |  |
|  | Unknown | 47 (48) | 32 (32.7) | 19 (19.4) |  |
| Duration of illness | <=4 Weeks | 54 (49.1) | 32 (29.1) | 24 (21.8) | 0.429 |
|  | >4 Weeks | 142 (53.6) | 80 (30.2) | 43 (16.2) |  |
| Level of Social Support | Poor Social Support | 49 (43.8) | 32 (28.6) | 31 (27.7) | 0.004 |
|  | Strong Social Support | 147 (55.9) | 80 (30.4) | 36 (13.7) |  |
| Medication Adherence | Adherent | 187 (55) | 98 (28.8) | 55 (16.2) | 0.002 |
|  | Non-Adherent | 9 (25.7) | 14 (40) | 12 (34.3) |  |

**Table S2.3: Chi-square test of association of anxiety with socio-demographic factors amongst TB patients in the Fako Division of Cameroon**

| **Variable** | | **Variable Response** | **Severity of Anxiety** | | | **P-Value** |
| --- | --- | --- | --- | --- | --- | --- |
|  |  |  | **Normal** | **Borderline Abnormal** | **Abnormal** |  |
|  |  |  | **N (%)** | **N (%)** | **N (%)** |  |
| Name of Hospital | | Buea Regional Hospital | 56 (45.5) | 37 (30.1) | 30 (24.4) | <0.001 |
|  |  | Baptist Hospital Mutengene | 128 (82.1) | 22 (14.1) | 6 (3.8) |  |
|  |  | Tiko Cottage Hospital | 21 (84) | 0 (0) | 4 (16) |  |
|  |  | Tiko District Hospital | 22 (73.3) | 6 (20) | 2 (6.7) |  |
|  |  | Limbe Regional Hospital | 36 (87.8) | 5 (12.2) | 0 (0) |  |
| Age Group | | 21-30 | 113 (71.1) | 29 (18.2) | 17 (10.7) | 0.479 |
|  |  | 31-40 | 90 (75.6) | 17 (14.3) | 12 (10.1) |  |
|  |  | 41-50 | 34 (58.6) | 15 (25.9) | 9 (15.5) |  |
|  |  | 51-60 | 10 (62.5) | 5 (31.3) | 1 (6.3) |  |
|  |  | >=61 | 16 (69.6) | 4 (17.4) | 3 (13) |  |
| Sex | | Female | 97 (65.5) | 31 (20.9) | 20 (13.5) | 0.275 |
|  |  | Male | 166 (73.1) | 39 (17.2) | 22 (9.7) |  |
| Marital Status | | Single | 167 (74.9) | 34 (15.2) | 22 (9.9) | 0.014 |
|  |  | Married | 89 (64) | 34 (24.5) | 16 (11.5) |  |
|  |  | Widow | 6 (60) | 2 (20) | 2 (20) |  |
|  |  | Widower | 1 (33.3) | 0 (0) | 2 (66.7) |  |
| Educational Level | | Primary | 67 (64.4) | 25 (24) | 12 (11.5) | 0.436 |
|  |  | Secondary | 157 (74.1) | 34 (16) | 21 (9.9) |  |
|  |  | Tertiary | 36 (65.5) | 11 (20) | 8 (14.5) |  |
|  |  | No Education | 3 (75) | 0 (0) | 1 (25) |  |
| Residence | Rural | 106 (70.2) | 31 (20.5) | 14 (9.3) | 0.52 |  |
|  | Urban | 157 (70.1) | 39 (17.4) | 28 (12.5) |  |  |
| Employment status | Employed | 128 (64.3) | 48 (24.1) | 23 (11.6) | 0.012 |  |
|  | Unemployed | 135 (76.7) | 22 (12.5) | 19 (10.8) |  |  |
| Occupation | Government Employed | 1 (14.3) | 3 (42.9) | 3 (42.9) | 0.026 |  |
|  | Merchant/Business | 75 (70.8) | 19 (17.9) | 12 (11.3) |  |  |
|  | Private Employed | 64 (64.6) | 26 (26.3) | 9 (9.1) |  |  |
|  | Farmer | 32 (68.1) | 8 (17) | 7 (14.9) |  |  |
|  | Housewife | 16 (76.2) | 3 (14.3) | 2 (9.5) |  |  |
| Religion | Presbyterian | 59 (72) | 14 (17.1) | 9 (11) | 0.787 |  |
|  | Muslim | 6 (75) | 1 (12.5) | 1 (12.5) |  |  |
|  | Atheist | 13 (65) | 6 (30) | 1 (5) |  |  |
|  | Catholic | 58 (69.9) | 17 (20.5) | 8 (9.6) |  |  |
|  | Pentecostals | 104 (68.4) | 30 (19.7) | 18 (11.8) |  |  |
|  | Baptist | 23 (76.7) | 2 (6.7) | 5 (16.7) |  |  |
| Living alone | No | 209 (68.1) | 63 (20.5) | 35 (11.4) | 0.122 |  |
|  | Yes | 54 (79.4) | 7 (10.3) | 7 (10.3) |  |  |
| Family Size | <=5 | 153 (70.8) | 43 (19.9) | 20 (9.3) | 0.338 |  |
|  | >5 | 110 (69.2) | 27 (17) | 22 (13.8) |  |  |
| Monthly Income | <50 000 | 26 (70.3) | 6 (16.2) | 5 (13.5) | 0.001 |  |
|  | 50 000 to 100 000 | 159 (72.6) | 36 (16.4) | 24 (11) |  |  |
|  | 100 001 to 200 000 | 34 (61.8) | 12 (21.8) | 9 (16.4) |  |  |
|  | 200 001 to 300 000 | 21 (91.3) | 2 (8.7) | 0 (0) |  |  |
|  | >300 000 | 3 (21.4) | 9 (64.3) | 2 (14.3) |  |  |
|  | No Income | 20 (74.1) | 5 (18.5) | 2 (7.4) |  |  |

**Table S2.4: Chi-square test of association of depression with Clinical, social support and medication adherence factors amongst TB patients in the Fako Division of Cameroon**

| **Variable** | **Variable Response** | **Severity of Anxiety** | | | **P-Value** |
| --- | --- | --- | --- | --- | --- |
|  |  | **Normal** | **Borderline Abnormal** | **Abnormal** |  |
|  |  | **N (%)** | **N (%)** | **N (%)** |  |
| Classification Type | Pulmonary TB | 235 (72.3) | 57 (17.5) | 33 (10.2) | 0.058 |
|  | Extrapulmonary TB | 28 (56) | 13 (26) | 9 (18) |  |
| Sputum Type | Positive | 213 (72) | 55 (18.6) | 28 (9.5) | 0.107 |
|  | Negative | 50 (63.3) | 15 (19) | 14 (17.7) |  |
| Patient Status | Hospitalized | 20 (47.6) | 11 (26.2) | 11 (26.2) | 0.001 |
|  | Outpatient | 243 (73) | 59 (17.7) | 31 (9.3) |  |
| Category of Treatment | New | 246 (71.9) | 60 (17.5) | 36 (10.5) | 0.328 |
|  | Default | 5 (45.5) | 4 (36.4) | 2 (18.2) |  |
|  | Retreatment | 8 (50) | 4 (25) | 4 (25) |  |
|  | Relapse | 2 (66.7) | 1 (33.3) | 0 (0) |  |
|  | Failure | 2 (66.7) | 1 (33.3) | 0 (0) |  |
| Substance Use | No | 225 (70.8) | 58 (18.2) | 35 (11) | 0.823 |
|  | Yes | 38 (66.7) | 12 (21.1) | 7 (12.3) |  |
| Family History of Mental Illness | No | 255 (71) | 67 (18.7) | 37 (10.3) | 0.031 |
|  | Yes | 8 (50) | 3 (18.8) | 5 (31.3) |  |
| HIV/TB Coinfection | No | 214 (71.6) | 53 (17.7) | 32 (10.7) | 0.482 |
|  | Yes | 49 (64.5) | 17 (22.4) | 10 (13.2) |  |
| MDR TB | No | 260 (70.3) | 68 (18.4) | 42 (11.4) | 0.391 |
|  | Yes | 3 (60) | 2 (40) | 0 (0) |  |
| Phase of treatment | Initiation Phase | 103 (65.6) | 31 (19.7) | 23 (14.6) | 0.148 |
|  | Continuation Phase | 160 (73.4) | 39 (17.9) | 19 (8.7) |  |
| BMI | Normal | 121 (73.8) | 33 (20.1) | 10 (6.1) | 0.002 |
|  | Underweight | 28 (57.1) | 8 (16.3) | 13 (26.5) |  |
|  | Overweight | 32 (74.4) | 9 (20.9) | 2 (4.7) |  |
|  | Obesity | 10 (47.6) | 6 (28.6) | 5 (23.8) |  |
|  | Unknown | 72 (73.5) | 14 (14.3) | 12 (12.2) |  |
| Duration of illness | <=4 Weeks | 66 (60) | 25 (22.7) | 19 (17.3) | 0.012 |
|  | >4 Weeks | 197 (74.3) | 45 (17) | 23 (8.7) |  |
| Level of Social Support | Poor Social Support | 65 (58) | 27 (24.1) | 20 (17.9) | 0.002 |
|  | Strong Social Support | 198 (75.3) | 43 (16.3) | 22 (8.4) |  |
| Medication Adherence | Adherent | 247 (72.6) | 57 (16.8) | 36 (10.6) | 0.003 |
|  | Non-Adherent | 16 (45.7) | 13 (37.1) | 1. (17.1) |  |
